# An Alternative Synthesis of 3′,4′-Diaminoflavones to Evaluate Their Antioxidant Ability and Cell Apoptosis of Zebrafish Larvae

**DOI:** 10.3390/molecules17078206

**Published:** 2012-07-09

**Authors:** Tzenge-Lien Shih, Chih-Ang Hsiao, Zi-Yu Lin, Yau-Hung Chen

**Affiliations:** Department of Chemistry, Tamkang University, Tamsui Dist., 25137 New Taipei City, Taiwan; Email: z90107@yahoo.com.tw (C.-A.H.); shes060215@yahoo.com.tw (Z.-Y.L.)

**Keywords:** antioxidant, Baker-Venkataraman rearrangement, diaminoflavones, ROS-scavenging, zebrafish larvae

## Abstract

We described herein a concise synthesis of 3′,4′-diaminoflavone **10**. This new, three-step synthetic approach is more efficient than the conventional seven-step synthetic method. The route is shortened significantly by introducing the amino moieties early and eliminating the need for nitro group reduction. The other two analogues, 5,7-dihydroxy-3′,4′-diaminoflavone **11** and 5,7-dimethoxy-3′,4′-diaminoflavone **12**, were also synthesized similarly. The above three compounds, along with flavone, were evaluated for their antioxidant and UVB-protection abilities on zebrafish larvae. The data showed that compound **10** exhibited the best result, with −102.3% of ROS-scavenging rate.

## 1. Introduction

Over 6,500 flavonoids are known [1], and many naturally occurring flavonoids show bioactivity [2,3]. Flavonoids are widely distributed in vegetables and plants [4,5,6], and their diverse biological roles have been extensively investigated. For example, many flavonoids show antioxidant activity [2,3,7], are metal chelators [8,9], cell-membrane protectors [10,11,12], or oxidase inhibitors [13]. Certain synthetic flavonoid analogues have shown to prevent inflammation [14,15], cancer [16,17,18,19], or cardiovascular diseases [20].

The hydroxyl groups presented in flavonoids play an important role in their activities [21]. It has been claimed that the amino groups in flavonoids have the same behaviors as hydroxyl groups as hydrogen bond donors and acceptors [22]. Therefore, replacement of the hydroxyl groups with amino groups in flavonoids may help to develop more soluble salt form of flavonoids while sustaining or even improving their biological activities [22].

Few natural or synthetic aminoflavonoids have been reported however [21,22,23,24,25,26,27,28,29,30,31,32]. The most common method includes the reduction of the nitro [23,24,26,28,29] or azido [27,32] group(s) on the aromatic rings, followed by protection and deprotection of the resulting amino moieties [25,28,30,31]. As a part of our ongoing interest in the various aminoflavonoids, we are interested in the roles of their amino groups. We have selected for study compounds **10**, **11** and **12**, which bear amino groups at the 3′ and 4′ positions in the B ring and various substituents (H, OH, OMe) at the A rings. We wished to understand more the roles of amino groups on the A ring of flavones to compare with the existing potent antioxidants, such as luteolin [2]. This should allow us to evaluate their antioxidant ability. Among these molecules, we have prepared in three steps (~24.7% total yield) compound **10**, which was previously synthesized by Göker *et al.* in seven steps (~11.5% total yield) [23]. Based on the same strategy, compounds **11** and **12** could be easily prepared in an efficient manner.

In order to rapidly screen the above diaminoflavones, we selected the zebrafish as an excellent model organism for chemical and toxicological studies because of its physiological similarity to mammals. In particular, the rapid developed and optical transparency of zebrafish embryos allow non-invasive cellular ROS detection *in vivo*. In this regard, the antioxidant abilities of flavone and newly synthesized diaminoflavones **10**, **11**, and **12** were evaluated on zebrafish larvae.

## 2. Results and Discussion

### 2.1. Chemistry

**Scheme 1:**
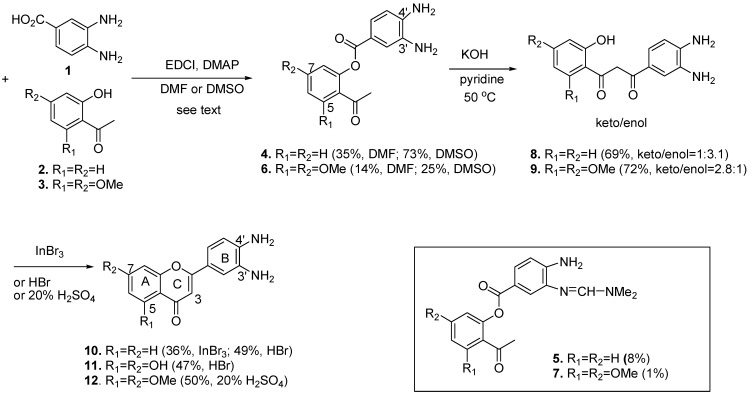
Syntheses of 3′,4′-diaminoflavone **10** and analogues **11** and **12**.

An altenative synthesis of 3,4-diaminoflavone **10** is depicted in Scheme 1. Unlike the early reported method for the synthesis of compound **10** [23], we chose commercially available free diamino compound **1** as the starting material. Compound **1** was coupled with **2** by EDCI in DMF to afford **4** in 35% yield. Although the DMF increased the solubility of starting materials, the lower yield was due to the formation of **5** (8%) and recovered unreacted **2** (53% conversion). Compound **5** was derived from the reaction of one of the amino groups of **4** with DMF. The structure elucidation of **5** was based on its HMQC and HMBC spectra. When DMF was replaced with DMSO, the coupling yield of **4** was enhanced significantly, up to 73%, and only small amounts of **2** were recovered (92% conversion). Compound **4** underwent smoothly the Baker-Venkataraman rearrangement [28,33,34,35] under KOH/pyridine conditions [28] at 50 °C to afford **8** in a keto/enol form (1/3.1) mixture. Compound **8** was treated with InBr_3_ (0.5 equiv.) [36] under reflux conditions to afford compound **9** in 36% yield. The yield was enhanced to 49% when **8** was heated under reflux condition in 48% HBr solution.

The same strategy was also employed for the synthesis of **11** which possesses two hydroxyl groups at C5 and C7 of the A ring. The two hydroxyl groups of 2′,4′,6′-trihydroxyacetophenone can be selectively protected [37] to furnish **3**, which was subjected to coupling with **1** to give **6** in 25% yield in DMSO (38% conversion of **3**). When DMF was used as solvent, compound **6** was isolated in 14% yield (22% conversion of **3**) and a trace amount of **7** (1%) was also formed. We found out the electron-donating groups in A ring affects the coupling yields. The Baker-Venkataraman rearrangement of **6** to **9** gave comparable yields as the preparation of **8**, but the keto/enol ratio was 2.8:1, which is opposite the result seen for **8** (1:3.1). Sequential cyclization and demethylation of compound **9** by heating in HBr afforded **11** in 47% yield. The best yield of compound **12** was isolated in 50% when compound **9** was heated under reflux in 20% H_2_SO_4_ solution.

### 2.2. Comparison of the ROS-Scavenging Ability of Flavone and Aminoflavones *10*, *11*, and *12*

**Figure 1:**
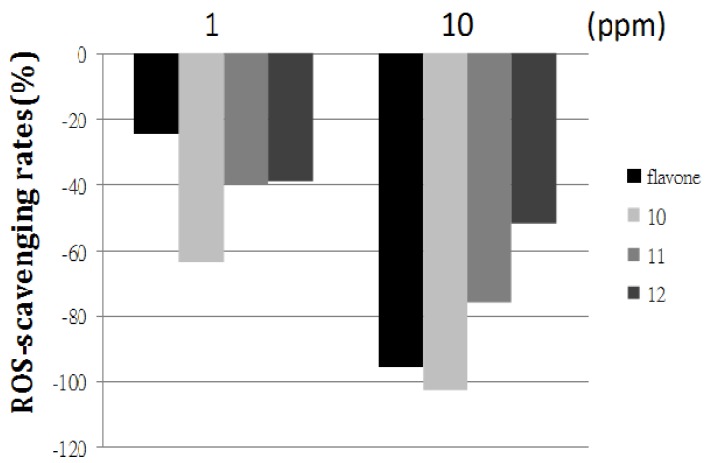
Comparison of ROS-scavenging ability of flavone and 3′,4′-diaminoflavones **10**, **11**, and **12**.

We have previously developed a protocol to detect the level of ROS-scavenaging in zebrafish embryos [38]. The same protocol was employed to evaluate the newly synthesized diaminoflavones **10**, **11**, and **12** with flavone for comparison. As shown in Figure 1, the detected ROS-scavenging rates in flavone-treated zebrafish embryos were decreased in a concentration dependent manner by −24.4% (1 ppm of flavone) and −94.5% (10 ppm of flavone) in comparison with that of the UV group (without addition of flavone). As previously described [38,39], negative ROS-scavenging rates indicated that the testing flavones possesses of ROS-scavenging activities. Similar dose-dependent results were obtained when zebrafish embryos were treated by different concentrations (1 and 10 ppm, individually) of diaminoflavones **10**, **11**, and **12** (ROS-scavenging rates: −39% to −102.3%). These data clearly demonstrated that diaminoflavone **10** exhibited the highest ROS-scavenging ability compared with those of flavone, **11** and **12** in low (1 ppm) as well as high concentrations (10 ppm).

### 2.3. Flavone and Diaminoflavones Protecting Zebrafish Cells from UV-Induced Apoptosis

**Figure 2:**
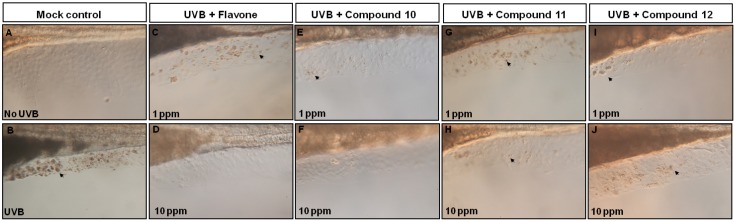
UVB exposure results for cell apoptosis in the fin region. Lateral views of mock control embryos without (**A**) and with UVB exposure (**B**) after TUNEL assay staining. (**C**, **E**, **G**, **I**) Lateral views of embryos derived from UVB + 1 ppm flavones group, or (**D**, **F**, **H**, **J**) UV+10 ppm flavones after TUNEL assay staining. Arrows indicate the apoptotic cells.

Zebrafish is an efficient model for evaluating sun-protective compounds because embryonic zebrafish fins are very sensitive to UVB radiation [38,39]. Using the zebrafish model, we have shown that flavone could protect zebrafish fins from UVB-induced apoptosis [40]. Therefore, we used the same protocol on flavone, diaminoflavones **10**, **11**, and **12** to compare with their UVB-protection ability. Results showed that no apoptotic signals were observed in the mock control embryos (no UVB, Figure 2A). After exposing fish to UVB, many apoptotic signals accompanying with malformed fin phenotypes were observed in the embryos (arrow in Figure 2B). However, few signals at 1 ppm or no signals at 10 ppm were found when those embryos were co-exposed to UVB with flavone or diaminoflavones **11** and **12** (Figure 2C,D,G–J). The concentrations of aminoflavone **10** at either 1 ppm or 10 ppm were enough protecting fin cells from apoptosis. We concluded that compound **10** might have the highest UVB-protection ability among these flavones (Figure 2E,F). On the basis of these observations, we suggest that flavone, diaminoflavones **10**, **11**, and **12** are able to protect UVB-damaged fin cells from apoptosis.

## 3. Experimental

### 3.1. General

Melting points were determined on a MP-2D apparatus and were not corrected. All chemicals were commercially available and used without further purification except otherwise mentioned. ^1^H (600 MHz) and ^13^C (150 MHz) NMR spectra were recorded on a Bruker 600 MHz instrument and the units were reported in *δ* (ppm). Mass spectra (LRMS and HRMS) were recorded on a Finnigan MAT 95S spectrometer. 

### 3.2. Synthetic Procedures for the Key Intermediates and Aminoflavones *10*, *11*, and *12*

*2′-(3,4-Diaminobenzoyloxy)acetophenone* (**4**). A solution of 3,4-diaminobenzoic acid (**1**, 1.264 g, 8.307 mmol) in DMSO (25 mL) was sequentially added 2′-hydroxyacetophenone (**2**, 0.50 mL, 4.154 mmol), EDCI (1.991 g, 10.385 mmol), and DMAP (0.254 g, 2.077 mmol) at 0 °C. This mixture was stirred for 17.5 h at ambient temperature. The reaction was diluted by Na_2_S_2_O_3_ (sat’d). The brown solid was filtered and the filtrate was extracted by EtOAc (×3). The organic layer was dried (MgSO_4_) and concentrated. Purification by flash column chromatography (200–350 mesh amino SiO_2_, hex/EtOAc = 2/1–1/2) afforded a dark red syrup and recovered **2** (0.105 g). This syrup was crystallized from a mixture of hex/EtOAc (v/v = 7/10) solution to give a pale yellow solid (0.820 g). Yield = 73%. MP = 156.5–158.5 °C. ^1^H-NMR (C_6_D_6_) *δ* 7.82 (d, *J* = 8.1 Hz, 1H), 7.65 (d, *J* = 7.9 Hz, 1H), 7.49 (s, 1H), 7.02 (d, *J* = 4.3 Hz, 2H), 6.84 (dt, *J* = 7.9, 4.3 Hz, 1H), 6.19 (d, *J* = 8.1 Hz, 1H), 2.99 (s, 2H), 2.44 (s, 2H), 2.27 (s, 3H). ^13^C-NMR (C_6_D_6_) *δ* 197.2, 165.6, 150.8, 142.2, 134.1, 133.2, 133.1, 126.0, 124.8, 124.7, 120.5, 119.5, 115.1, 39.2. HRMS (ESI) ([M^+^]) calcd for C_15_H_14_N_2_O_3_ 270.1004. Found: 270.1002.

*2′-Acetylphenyl-2-(dimethylamino)-1H-benzo[d]imidazole-6-carboxylate* (**5**). Purification by flash column chromatography (230−400 mesh SiO_2_, hex/EtOAc = 2/1–0/1) provided a white solid. Yield = 8%. Mp = 148.0–151.0 °C. ^1^H-NMR (CDCl_3_) *δ* 7.82 (dd, *J* = 7.7, 1.2 Hz, 1H), 7.75 (dd, *J* = 8.2, 1.8 Hz, 1H), 7.69 (s, 1H), 7.56 (d, *J* = 1.7 Hz, 1H), 7.54 (dd, *J* = 8.6, 1.5 Hz, 1H), 7.31 (t, *J* = 7.4 Hz, 1H), 7.21 (d, *J* = 8.1 Hz, 1H), 6.70 (d, *J* = 8.2 Hz, 1H), 4.65 (br s, 2H), 3.05 (s, 6H), 2.54 (s, 3H). ^13^C-NMR (CDCl_3_) *δ* 198.2, 165.4, 153.1, 150.0, 146.7, 137.6, 133.1, 132.0, 129.9, 126.9, 126.1, 124.0, 119.1, 117.8, 112.9, 40.3, 34.5, 30.4. HRMS (EI) ([M^+^]) calcd for C_18_H_17_N_3_O_3_ 325.1426. Found: 325.1429.

*1-(2-Hydroxyphenyl)-3-[3′,4′-diamino]propane-1,3-dione* (**8**). To a solution of **4** (0.539 g, 1.993 mmol) in pyridine (10 mL) was added KOH (0.168 g, 2.989 mmol) and the mixture was stirred at 50 °C for 3 h. The reaction was adjusted to pH 3 by 2 N HCl and extracted with EtOAc (×3). The organic layer was dried (MgSO_4_), filtered through celite, and concentrated. Purification by flash column chromatography (230–400 mesh SiO_2_, hex/EtOAc = 2/1–0/1) afforded a red solid (0.369 g). Yield = 69%. MP = 168.0–170.0 °C. (major, enol form): ^1^H-NMR (CDCl_3_) *δ* 7.95 (d, *J* = 7.9 Hz, 1H), 7.41 (t, *J* = 7.7 Hz, 1H), 7.29 (d, *J* = 8.3 Hz, 1H), 7.25 (s, 1H), 7.08 (s, 1H), 6.97−6.90 (m, 2H), 6.58 (d, *J* = 8.3 Hz, 1H), 5.61 (NH_2_), 4.74 (br s, NH_2_). (minor, keto form). ^1^H-NMR (CDCl_3_) *δ* 7.80 (d, *J* = 7.9 Hz, 1H), 7.50 (t, *J* = 7.7 Hz, 1H), 7.19 (d, *J* = 8.2 Hz, 1H), 7.14 (s, 1H), 6.97–6.90 (m, 2H), 6.53 (d, *J* = 8.1 Hz, 1H), 5.52 (NH_2_), 4.59 (br s, NH_2_). (major, enol form). ^13^C-NMR (CDCl_3_) *δ* 201.1, 185.7, 183.4, 159.2, 141.7, 134.1, 134.0, 129.0, 121.4, 120.0, 119.5, 119.2, 117.5, 112.9, 112.5, 92.7. (minor, keto form). ^13^C-NMR (CDCl_3_) *δ* 192.2, 160.5, 136.1, 133.9, 131.3, 125.3, 120.9, 117.6, 113.5, 112.2, 50.1. HRMS (ESI) ([M^+^]) calcd for C_15_H_14_N_2_O_3_ 270.1004. Found: 270.1002.

*2-(3,4-Diaminophenyl)-4H-1-benzopyran-4-one* (**10**). A solution of compound **8** (0.110 g, 0.407 mmol) was dissolved in 48% HBr (5 mL) and heated under reflux for 15 h. The mixture was slowly poured into Na_2_S_2_O_3_ saturated solution and extracted with EtOAc (×3). The organic layer was dried (MgSO_4_) and concentrated. Purification by flash column chromatography (230–400 mesh SiO_2_, hex/EtOAc = 1/1–0/1) furnished a red solid which was washed several times with methanol to give a yellow-red solid (0.050 g). Yield = 49%. MP = 256.0–258.0 °C. *lit.*^23^ 237 °C (dec.). ^1^H-NMR (DMSO-*d*_6_) *δ* 8.00 (d, *J* = 7.7 Hz, 1H), 7.77 (t, *J* = 7.4 Hz, 1H), 7.65 (d, *J* = 8.3 Hz, 1H), 7.45 (t, *J* = 7.4 Hz, 1H), 7.23 (d, *J* = 8.4 Hz, 1H), 7.22 (s, 1H), 6.62 (d, *J* = 7.9 Hz, 1H), 6.59 (s, 1H), 5.39 (s, 2H, NH_2_), 4.75 (s, 2H, NH_2_). ^13^C-NMR (DMSO-*d*_6_) *δ* 176.5, 164.5, 155.5, 140.0, 134.6, 133.8, 125.1, 124.7, 123.5, 118.3, 118.0, 117.3, 113.5, 111.4, 103.0. HRMS (ESI) ([M^+^]) calcd for C_15_H_12_N_2_O_2_ 252.0899. Found: 252.0899.

*4′,6′-Dimethoxy-2′-(3,4-diaminobenzoyloxy)acetophenone* (**6**). Compound **1** (3.726 g, 24.486 mmol) and compound **3** (2.826 g, 14.402 mmol) were dissolved in DMSO (85 mL) at 0 °C. To this cold solution was added EDCI (5.522 g, 28.804 mmol) and DMAP (1.231 g, 10.083 mmol) and stirred for 48 h. The reaction was added Na_2_S_2_O_3_ saturated solution. The resulting brown solid was filtered and the filtrant was extracted by EtOAc (×3). The organic layer was dried (MgSO_4_), concentrated, and purified by flash column chromatography (230–400 mesh SiO_2_, hex/EtOAc = 2/1–1/2) to provide an orange solid (1.192 g) and recovered **3** (1.760 g). Yield = 25%. MP = 174.0–176.0 °C. ^1^H-NMR (CDCl_3_) *δ* 7.56 (dd, *J* = 8.2, 1.9 Hz, 1H), 7.47 (d, *J* = 1.9 Hz, 1H), 6.68 (d, *J* = 8.2 Hz, 1H), 6.37 (d, *J* = 2.2 Hz, 1H), 6.34 (d, *J* = 2.2 Hz, 1H), 3.84 (s, 3H), 3.80 (s, 3H), 2.46 (s, 3H). ^13^C-NMR (CDCl_3_) *δ* 199.8, 165.0, 162.0, 158.8, 149.9, 141.5, 133.1, 124.3, 119.5, 118.9, 117.7, 114.8, 100.1, 96.4, 55.9, 55.6, 31.9. HRMS (ESI) ([M^+^]) calcd for C_17_H_18_N_2_O_5_ 330.1216. Found: 330.1214.

*2-Acetyl-3,5-dimethoxyphenyl-2-(dimethylamino)-1H-benzo[d]imidazole-6-carboxylate* (**7**). The same procedure as in preparation of **5** was used to give a white solid. MP = 58.0–62.0 °C. ^1^H-NMR (CDCl_3_) *δ* 7.72–7.68 (br d, 2H), 7.54 (s, 1H), 6.67 (d, *J* = 8.3 Hz, 1H), 6.37 (d, *J* = 2.1 Hz, 1H), 6.36 (d, *J* = 2.1 Hz, 1H), 3.84 (s, 3H), 3.81 (s, 3H), 3.10 (s, 6H), 2.46 (s, 3H). ^13^C-NMR (CDCl_3_) *δ* 199.9, 165.3, 161.9, 158.6, 153.1, 150.0, 146.5, 137.4, 127.0, 119.1, 118.0, 117.9, 112.9, 100.1, 96.4, 55.9, 55.6, 40.3, 34.5, 31.9. HRMS (EI) ([M^+^]) calcd for C_20_H_21_N_3_O_5_ 385.1638. Found: 385.1631

*1-(3,4-Diaminophenyl)-3-(2-hydroxy-4,6-dimethoxyphenyl)propane-1,3-dione* (**9**). The same procedure as in preparation of **8** was used. Purification by flash column chromatography (230–400 mesh SiO_2_, hex/EtOAc = 2/1–0/1) provided a pale orange solid. Yield = 72%. MP = 207.0−209.0 °C. (major, keto form): ^1^H-NMR (DMSO-*d*_6_) *δ* 13.76 (s, 1H), 7.16–7.12 (m, 2H), 6.54 (d, *J* = 8.1 Hz, 1H), 6.10 (d, *J* = 2.3 Hz, 1H), 6.00 (d, *J* = 2.3 Hz, 1H), 5.45 (s, 2H), 4.68 (br s, 2H), 4.38 (s, 2H), 3.80 (s, 3H), 3.44 (s, 3H). (minor, enol form): ^1^H NMR (DMSO-*d*_6_) *δ* 12.75 (s, 1H), 7.12–7.10 (m, 1H), 6.98 (s, 1H), 6.57 (d, *J* = 8.8 Hz, 1H), 6.14 (d, *J* = 2.2 Hz, 1H), 6.08 (d, *J* = 2.2 Hz, 1H), 5.55 (s, 2H), 4.77 (br s, 2H), 3.90 (s, 3H), 3.79 (s, 3H) (major, keto form). ^13^C-NMR (DMSO-*d*_6_) *δ* 200.8, 192.5, 166.5, 166.2, 162.2, 141.3, 133.8, 125.4, 120.3, 113.3, 112.3, 105.3, 93.8, 90.7, 55.8, 55.7, 53.9 (minor, enol form). ^13^C-NMR (DMSO-*d*_6_) *δ* 188.6, 179.5, 164.1, 163.8, 160.9, 134.1, 120.8, 118.6, 113.1, 112.1, 104.3, 95.3, 94.2, 91.0, 56.1, 55.5. HRMS (ESI) ([M^+^]) calcd for C_17_H_18_N_2_O_5_ 330.1216. Found: 330.1213. 

*2-(3,4-Diaminophenyl)-5,7-dihydroxy-4H-chromen-4-one* (**11**). The same procedure as in preparation of **10** was used.Purification by flash column chromatography (230–400 mesh SiO_2_, hex/EtOAc = 2/1–0/1) provided an orange-yellow solid. The resulting solid was repeatedly washed with MeOH to afford a pale orange-yellow solid. Yield = 47%. MP = 323 °C (dec.). ^1^H-NMR (DMSO-*d*_6_) *δ* 13.09 (-OH), 7.17–7.14 (m, 2H), 6.59 (d, *J* = 8.1 Hz, 1H), 6.44 (s, 1H), 6.33 (d, *J* = 1.6 Hz, 1H), 6.09 (d, *J* = 1.6 Hz, 1H), 5.40 (s, 2H, -NH_2_), 4.73 (br s, 2H, -NH_2_). ^13^C-NMR (DMSO-*d*_6_) *δ* 181.2, 165.1, 164.9, 161.4, 157.3, 140.2, 134.5, 117.9, 117.4, 113.5, 111.2, 103.1, 100.7, 98.9, 93.8. HRMS (ESI) ([M^+^]) calcd for C_15_H_12_N_2_O_4_ 284.0797. Found: 284.0792.

*2-(3,4-Diaminophenyl)-5,7-dimethoxy-4H-chromen-4-one* (**12**). Compound **9** was heated under reflux in 20% H_2_SO_4_ for 12 h. The reaction mixture was slowly poured into a cold saturated NaHCO_3_ solution. The mixture was extracted with EtOAc, dried (MgSO_4_) and purified by flash column chromatography (230–400 mesh SiO_2_, hex/EtOAc = 1/2−0/1) to provide an orange-red solid. Yield = 50%. MP = 152.0–154.0 °C. ^1^H-NMR (DMSO-*d*_6_) *δ* 7.13–7.11 (m, 2H), 6.69 (d, *J* = 2.3 Hz, 1H), 6.58 (d, *J* = 8.7 Hz, 1H), 6.45 (d, *J* = 2.3 Hz, 1H), 6.29 (s, 1H), 5.25 (s, 2H), 4.68 (s, 2H), 3.87 (s, 3H), 3.80 (s, 3H). ^13^C-NMR (DMSO-*d*_6_) *δ* 175.5, 163.3, 161.5, 160.2, 159.1, 139.4, 134.5, 118.1, 116.6, 113.5, 111.1, 108.3, 104.5, 96.0, 93.1, 56.0, 55.9. HRMS (EI) ([M^+^]) calcd for C_17_H_16_N_2_O_4_ 312.1110. Found: 312.1110.

### 3.3. Evaluation

#### 3.3.1. Methods for Fish Embryos Maintenance, Chemicals Treatment and Survival Rates Analysis

The procedures for zebrafish culture and embryo collection used in this study have been described previously [41,42]. For chemicals treatment, flavone and diaminoflavones **10**, **11** and **12** were individually dissolved in DMSO to the designated concentrations (1, and 10 ppm). Thirty embryos were collected and treated with different concentrations of aminoflavones for 3 h (72−75 hpf) then counted for their survival rates.

#### 3.3.2. UVB Exposure, ROS Detection and Data Analysis

Modified procedures were used in this study for UVB exposure and embryo collection [38,39]. After UVB exposure, all embryos were cultivated in 6-well cell culture plates until the analysis of their ROS levels. To detect the accumulation of ROS in zebrafish embryos, embryos from the UVB-only group (no flavone added), UVB + flavone and embryos from the UVB + diaminoflavone groups (**10**, **11**, and **12**) were incubated with 500 ng/mL dihydrodichlorofluorescein diacetate (H_2_DCFDA, Molecular Probes, Eugene, OR, USA). After a 150 min incubation period at 28 °C, the fluorescence intensity of the embryo was measured at excitation/emission = 485/530 nm. All data were presented as “ROS-scavenging rates” calculated by the equation described previously [43]. A positive ROS-scavenging rate indicates that the treatment with the flavone leads to the generation of ROS. A negative ROS-scavenging rate means that the tested flavone compound exhibits ROS-scavenging activities.

## 4. Conclusions

In conclusion, we have developed an improved three-step synthesis of **10**. The same procedure was used to prepare two new compounds **11** and **12**. It is worth noting that the solvent plays an important role in the coupling yields, whereby DMSO was shown to be superior to DMF as solvent. In particular, compounds **10**, **11** and **12** have shown increased ROS scavenging ability along with UV-protecting abilities to compare with flavone. In this article, we established a rapid screening model of diaminoflavones by zebrafish larvae. We are also synthesizing a series of derivatives with diamino groups on the A ring of flavones along with compounds **10**–**12** to compare their bioactivities. The details will be published in due course. We conclude that the diaminoflavones have potential to develop as antioxidants and drugs.
